# Fine-scale dietary changes between the breeding and non-breeding diet of a resident seabird

**DOI:** 10.1098/rsos.140291

**Published:** 2015-04-08

**Authors:** Nicole D. Kowalczyk, André Chiaradia, Tiana J. Preston, Richard D. Reina

**Affiliations:** 1 School of Biological Sciences, Monash University, Clayton, Victoria 3800, Australia; 2 Research Department, Phillip Island Nature Parks, PO Box 97, Cowes, Victoria 3922, Australia

**Keywords:** isotopic niche, life-stages, seabird, opportunist, stable isotopes, anchovy

## Abstract

Unlike migratory seabirds with wide foraging ranges, resident seabirds forage in a relatively small range year-round and are thus particularly vulnerable to local shifts in prey availability. In order to manage their populations effectively, it is necessary to identify their key prey across and within years. Here, stomach content and stable isotope analyses were used to reconstruct the diet and isotopic niche of the little penguin (*Eudyptula minor*). Across years, the diet of penguins was dominated by anchovy (*Engraulis australis*). Within years, during winter, penguins were consistently enriched in *δ*^15^N and *δ*^13^C levels relative to pre-moult penguins. This was probably due to their increased reliance on juvenile anchovies, which dominate prey biomass in winter months. Following winter and during breeding, the *δ*^13^C values of penguins declined. We suggest this subtle shift was in response to the increased consumption of prey that enter the bay from offshore regions to spawn. Our findings highlight that penguins have access to both juvenile fish communities and spawning migrants across the year, enabling these seabirds to remain in close proximity to their colony. However, annual fluctuations in penguin isotopic niche suggest that the recruitment success and abundance of fish communities fluctuate dramatically between years. As such, the continued monitoring of penguin diet will be central to their ongoing management.

## 2. Introduction

Unlike most seabird species that undertake annual migrations, resident seabirds remain in close proximity to their breeding areas throughout the year. They depend on local resources during both the breeding and non-breeding seasons [1]. The availability and abundance of resources during the breeding season can shape their breeding events (e.g. lay date) and determine breeding success [2–4]. By contrast, resources obtained during the non-breeding season are critical to the successful completion of moult [1,5,6] and surviving the environmental constraints imposed by winter [7], and can have carry-over effects that influence subsequent breeding performance [8–10]. Therefore, to ensure their survival and reproductive success, resident seabirds require access to relatively predictable and local prey resources year-round.

As adults and chicks are most accessible for study during the breeding season, most seabird dietary studies (including resident seabird species) are undertaken at this life-stage [11]. These studies have been crucial in identifying particular prey species or age classes of prey that influence breeding performance and require protection [12–14]. By contrast, few studies have identified important foraging locations or resources necessary for the survival of seabirds during their non-breeding season, with most of these having focused on seabird winter diets [15,16]. This is primarily due to the logistical difficulties of tracking the diet of migratory or widely dispersing seabirds. Surprisingly, despite the year-round presence of resident seabird species, few studies have assessed their diet in the non-breeding season. The few existing studies comparing life-stage dietary shifts have found varying results. For example, in the Isle of May, Scotland, fatty acid analysis demonstrated that the pre-breeding and breeding diet of common guillemots (*Uria aalge*) differ substantially [17]. But, due to insufficient dietary analysis in the non-breeding season, the non-breeding diet of guillemots remains unknown [17]. By contrast, the winter and breeding diet of yellow-legged gulls (*Larus michahellis*) in the Bay of Biscay differ slightly, in a consistent manner across years, and this is attributed to seasonal fluctuations in resource availability [18]. These studies highlight that the breeding and non-breeding diets of resident seabirds differ, and that in order to manage their populations effectively it is necessary to have an understanding of their trophic relationships at all stages of the annual cycle. This is especially important for resident species that have a small foraging range and that are particularly vulnerable to local shifts in prey availability.

Stable isotope analyses of seabird tissues, in combination with conventional assessments of diet, are powerful tools to investigate the year-round foraging ecology of seabirds [19–21]. Recent advancements in isotope ecology have provided the statistical frameworks to reconstruct the diet of individuals or groups at specific temporal scales [22,23]. Consequently, the stable isotope ratios of consumers and their prey can be used in stable isotope mixing models to estimate the proportion of each prey group in the diet of consumers. Additionally, stable isotopes can be used to calculate the ‘isotopic niche’ of seabirds and provide quantitative information on resource and habitat use, parameters that can be used as proxies to define the ecological niche of populations [24,25].

Little penguins (*Eudyptula minor*) are resident seabirds with one of the shortest foraging ranges among seabird species [26,27], and local fluctuations in prey availability strongly influence their foraging and reproductive ecology [28]. The St Kilda penguin colony are thought to forage exclusively within Port Phillip Bay [29,30] on a predominantly clupeoid-based diet [31]. Their short foraging range and narrow dietary breadth make them particularly vulnerable to changes in the distribution and abundance of their prey [30]. In this study, little penguins were used as a model species to assess how the diets of small home-range, resident seabirds fluctuate between life-stages and years. Specifically, we assessed if these residents display inter-annual dietary and isotopic niche variation and evaluated if they display shifts in diet and isotopic niche between the pre-moult, winter and breeding stages of the annual cycle.

## 3. Material and methods

### 3.1 Study area and species

Fieldwork was carried out at the St Kilda breakwater, within Port Phillip Bay, Victoria, Australia (37°51′ S, 144°57′ E) over four years (2007, 2008, 2011 and 2012). This temperate, semi-enclosed tidal embayment is joined to Bass Strait through a 3-km-wide, shallow, channel [32]. The embayment has an approximate 1930 km^2^ area, with a mean depth of 13.6 m [33]. The St Kilda breakwater is located in the north of Port Phillip Bay and is occupied by approximately 1000 little penguins who reside on the breakwater year-round (Z. Hogg 2006, unpublished data). The annual cycle of little penguins comprises the non-breeding (moult and winter) and breeding seasons. During pre-moult (approx. February to March), adults accumulate sufficient reserves to sustain them during their annual moult. Little penguins fast ashore during moult and the moulting cycle lasts approximately 17 days [34]. After moult, adults return to sea and regain lost energy reserves. They return to the colony throughout the winter period (approx. March to September) and increase the time spent at the colony in preparation for the breeding season [35]. The commencement date of the breeding season is highly variable (May to September) both within and between colonies from year to year, but peak breeding occurs in the austral spring [36]. Typically, females lay one clutch of two eggs but have been recorded to lay up to three clutches in a season [37]. Males and females share the task of egg incubation, which spans approximately five weeks, and once chicks hatch they are brooded by at least one parent for two to three weeks, a period termed ‘guard’ [38]. After the ‘guard’ stage, both parents forage at sea, leaving the nest unguarded during the day and return to feed the chicks at night, a period termed ‘post-guard’. Chicks fledge at approximately eight weeks of age [28]. In this study, the incubation and guard stages of the breeding season are hereafter referred to as the ‘breeding life-stage’ as they comprise most of the breeding season.

### 3.2 Stomach content analysis

Ten penguins were caught when entering the St Kilda breakwater each month between February 2007 and October 2008. Individual penguins were identified via passive integrated transponders (Trovan, Ltd., Australia), and stomach contents from the sampled penguins were obtained using a modified water offloading technique [39] and frozen prior to analysis. Prey items were measured to the nearest 0.1 mm and identified from otoliths and squid beaks using methods outlined previously [31]. The length and weight of each fish and squid were calculated from published regression equations of otoliths and beaks, respectively [40–42]. Anchovy were categorized into broad age classes according to size, based on data from anchovies collected within Port Phillip Bay [43]. The less than 1-year cohort corresponded to standard lengths less than 63 mm, 1–2-year cohort 63–91 mm and 2–3-year cohort 92+ mm (none larger than 98 mm was recorded), recognizing that there is overlap in size between the age cohorts. No stomach analysis was conducted after October 2008 due to monthly consistencies in stomach content prey items.

Stomach content samples were quantified using a modified weighted relative occurrence method [44]. The percentage contribution of each item to the stomach sample based on mass (calculated by linear regression) was determined and averaged across all samples to provide a percentage contribution value for each prey item for each month [31].

### 3.3 Penguin tissue collection and preparation for stable isotopes

For animals of similar mass as little penguins (approx. 1 kg), the half-life of *δ*^13^C and *δ*^15^N stable isotopes in whole blood is 10–23 days [45]. Accordingly, in 2007 and 2008, individual penguins were identified via passive integrated transponders (Trovan, Ltd.) and a single blood sample was collected to represent the dietary intake of birds at either the pre-moult, winter or breeding (incubation and guard) stage of the annual cycle (table 1). Approximately 80 μl of blood was collected from the tarsal vein of adults using venipuncture and capillarity. Blood samples were stored in 70% ethanol at room temperature until analysis. Ethanol-based blood preservation does not appear to change stable carbon and nitrogen readings [46].

**Table 1.** Mean (±s.d.) values of stable carbon (*δ*^13^C) and nitrogen (*δ*^15^N) isotopes with corresponding C:N mass ratios from blood and feather (non-corrected and corrected) samples of adult penguins, at key life-stages (pre-moult, winter and breeding) over four years. Superscript letters indicate significantly different results within years based on Tukey's post hoc comparisons.

year	tissue	life-stage	date tissues obtained	*n* m, f	*δ*^13^C (‰)	*δ*^13^C (‰) corrected	*δ*^15^N (‰)	*δ*^15^N (‰) corrected	C:N mass ratio
2007	blood	pre-moult	Feb, Mar, Apr	18, 18	−19.1±0.4^a^		17.8±0.9^a^		3.2±0.1
	blood	winter	May, Jun, Jul	18, 17	−19.0±0.3^a^		18.4±0.5^b^		3.2±0.1
	blood	breeding	Sep, Oct, Nov	18, 18	−19.1±0.2^a^		19.1±0.4^c^		3.4±0.1
2008	blood	pre-moult	Feb, Mar, Apr	18, 18	−18.8±0.2^a^		18.2±0.6^a^		3.4±0.2
	blood	winter	May, Jun, Jul	18, 18	−18.5±0.3^b^		18.8±0.7^b^		3.3±0.8
	blood	breeding	Aug, Sep, Oct	18, 18	−19.0±0.5^a^		18.7±0.5^b^		3.2±0.2
2011	feather	pre-moult	Feb, Mar, Apr	21, 15	−18.2±0.6	−19.8±0.6^a^	19.8±1.6	19.1±1.6^a^	3.4±0.15
	blood	winter	May, Jun, Jul, Aug	25, 15	−17.6±0.5^b^		21.1±0.5^b^		3.4±0.27
	blood	breeding	Aug, Sep, Oct, Nov	20, 19	−18.7±0.4^c^		20.6±0.6^b^		3.4±0.1
2012	feather	pre-moult	Feb, Mar, Apr	20, 16	−18.0±0.4	−19.5±0.4^a^	20.2±1.4	19.4±1.4^a^	3.5±0.12
	blood	winter	May, Jun, Jul, Aug	19, 13	−18.3±0.6^b^		20.3±0.6^b^		3.5±0.11
	blood	breeding	Sep, Oct, Nov	21, 21	−19.1±0.6^c^		18.2±1.4^c^		3.5±0.16

In 2011 and 2012, blood samples were collected to represent winter and breeding dietary intake (table 1). Approximately 150 μl of blood was collected from the tarsal vein using venipuncture and capillarity and was then transferred onto a microscope slide and dried at ambient air temperature [47]. To provide dietary information on the pre-moult diet of penguins in 2011 and 2012, a feather sample was collected from the lower back of post-moult adults. As little penguins replace their feathers during their three-week fast ashore and because feathers are metabolically inert after growth, feathers are thought to reflect food consumed during the pre-moult foraging bout [48,49].

In the laboratory, blood samples were freeze-dried; blood lipids were not extracted prior to analysis given that the lipid component of blood is less than 1% of the total wet mass of whole blood [50]. Blood samples were powdered, loaded into tin capsules (8×5 mm), weighed (0.4–0.6 mg) and sealed. Entire adult feathers were washed with distilled water before being freeze-dried and finely cut using stainless steel scissors. Surface lipids were not removed using a chloroform/methanol solution because this process has been shown to have negligible effects on isotope ratios [51]. Feathers were homogenized, and a subsample of the entire feather was loaded into a tin capsule (8×5 mm), weighed (0.4–0.6 mg) and sealed.

### 3.4 Prey collection and preparation for stable isotopes

In 2007 and 2008, muscle tissue from anchovy (*Engraulis australis*), southern garfish (*Hyporhamphus melanochir*) and bay squid (*Loliolus noctiluca*) was collected from St Kilda penguin stomach contents for stable isotope analysis (table 2). These species were selected for stable isotope analysis due to their dominant presence in penguin stomach contents. Additional anchovy (*n*=23) and southern garfish (*n*=10) samples were obtained from commercial fishing boats that operate within Port Phillip Bay in the winter of 2008 (table 2). Anchovy were categorized into three ontogenetic stages based on their morphometric measurements, as described above [43]. Ontogenetic categories included: (i) less than 1 year class (*n*=6), (ii) 1–2 year class (*n*=8) and (iii) 2–3 year class (*n*=9). Due to the small sample size of prey in 2007 and the similar isotope ratios within species across years, prey items collected in 2007 and 2008 were pooled to reconstruct the 2007 and 2008 diet of penguins, similar to procedures adopted previously [52]. Prey items collected in these years are hereafter referred to as 2007/2008 prey.

**Table 2.** Mean (±s.d.) values of stable carbon (*δ*^13^C) (normalized and lipid removed values provided) and nitrogen (*δ*^15^N) isotopes with corresponding C:N mass ratios from fish samples obtained through either stomach content analysis or from fishing vessels within Port Phillip Bay over four years.

year	species	*n*	date collected	*δ*^13^C (‰)	*δ*^13^C (‰) normalized/lipid removed	*δ*^15^N (‰)	C:N mass ratio
2007	anchovy (*Engraulis australis*)	1^a^	Sep	−20.5	−21.2	15.69	3.5
	bay squid (*Loliolus noctiluca*)	1^a^	Dec	−19.6	−18.8	17.21	3.57
2008	anchovy (*E. australis*)	4^a^, 23^b^	Feb–Sep	−19.9±0.4	−20.5±0.4	15.4±1.1	3.3±0.1
	bay squid (*L. noctiluca*)	3^a^	Sep–Oct	−19.4±0.3	−18.6±0.3	18.3±1.7	3.8±0.3
	southern garfish (*Hyporhamphus melanochir*)	4^a^, 10^b^	Jun–July	−17.4±1.6	−17.8±1.6	16.3±0.9	3.3±0.8
2011	anchovy (*E. australis*)	5^b^	Sep	−18.9±0.4	−18.3±0.5	18.4±1.2	3.7±0.2
	southern garfish (*H. melanochir*)	5^b^	Sep	−17.2±0.9	−16.6±0.8	16.5±0.3	3.5±0.6
2012	anchovy (*E. australis*)	4^b^	Oct	−18.8±0.3	−18.0±0.1	18.0±0.5	3.8±0.2
	southern garfish (*H. melanochir*)	5^b^	Sep	−16.0±1.4	−15.8±1.5	16.2±1.5	3.5±0.1
	sandy sprat (*Hyperlophus vittatus*)	10^b^	Sep	−19.9±0.7	−20.5±0.7	15.4±1.4	3.4±0.05
	blue sprat (*Spatelloides robustus*)	3^b^	Sep	−20.9±0.8	−21.07±0.3	16.8±0.7	3.1±0.2
	juvenille pilchard (*Sardinops sagax*)	10^b^	Aug	−21.1±1.2	−20.0±0.2	11.5±0.3	4.2±0.2
	adult pilchard (*S. sagax*)	10^b^	Oct	−20.0±0.4	−18.5±0.2	16.5±0.6	4.3±0.4

^a^Collected from stomach content.

^b^Prey were obtained from fishing vessels that operate in Port Phillip Bay.

In 2011, anchovy and southern garfish (bay squid was not available) were obtained from commercial fishing boats that operate within Port Phillip Bay (table 2). In 2012, in addition to anchovy and southern garfish, sandy sprat (*Hyperlophus vittatus*), blue sprat (*Spatelloides robustus*) and pilchard (*Sardinops sagax*) were obtained from commercial fishing boats that operate within Port Phillip Bay (table 2). Clupeoids collected in 2011 and 2012 were collected for stable isotope analysis due to their increased abundance in Port Phillip Bay in 2011 [53], and were thus a potential prey source for little penguins as they have been found in the stomach contents of penguins at St Kilda and elsewhere [31,39,54]. Pilchards were categorized into juvenile and adult age classes in accordance with length-frequency data for pilchards obtained from commercial catches in Port Phillip Bay [55]. Size variations in other prey sources were not distinct, therefore these species were not separated into age classes. As all potential prey species were not collected in both 2011 and 2012, prey species were pooled across years so as to provide a wider range of dietary sources within mixing models. Prey obtained in 2011 and 2012 are hereafter referred to as 2011/2012 prey.

In 2007/2008, a section of prey caudal muscle was prepared for *δ*^13^C and *δ*^15^N stable isotope analysis. Lipids were not extracted prior to analysis. Samples were freeze-dried, ground, and 0.4–0.6 mg tissue samples were loaded into tin caps prior to stable isotope analysis. In 2011/2012, a section of the caudal muscle of prey samples was rinsed in deionized water and dried at 60°C in a glass vial until it reached a constant weight. Dried samples were ground and two samples were obtained from each vial; one was immediately prepared for stable isotope analysis (samples were freeze-dried, ground and loaded into tin caps) and the second underwent lipid extraction [56,57]. To remove lipids, samples were placed in glass centrifuge tubes and submerged in 2:1 *chloroform*:*methanol* solution. Samples were stirred and centrifuged for 10 min at 1318*g*. The supernatant containing solvent and lipids was removed. This process was repeated until the supernatant solvent was clear and colourless after centrifugation. Samples were then dried at 60°C for 24 h. Treated samples were freeze-dried, ground and 0.4–0.6 mg tissue samples were loaded into silver caps prior to stable isotope analysis.

Lipid extraction can induce shifts in isotope ratios (particularly *δ*^13^C values) [57], and the effects of lipid extraction are greatest on tissues when their C:N ratios are more than 4.0 [58]. The C:N ratios in penguin prey in 2007/2008 were at times more than 4.0 and therefore were anticipated to have some influence on the SIAR reconstructed diet of penguins. To accommodate differences in lipid extraction protocols between years 2007/2008 and 2011/2012, 2007/2008 prey *δ*^13^C values were normalized. Values were normalized by detracting the mean difference between lipid extracted and non-extracted *δ*^13^C values from 2011/2012 anchovy and southern garfish samples. As no squid were collected in 2011 and 2012, the mean difference between lipid extracted and non-extracted *δ*^13^C values could not be determined. We therefore used published values for squid to normalize data [59]. Normalized values were used for all statistical analyses.

### 3.5 Stable isotope analysis

In 2007 and 2008, samples were processed at the Stable Isotopes in Nature Laboratory, Canada, and were combusted in an AS128 autosampler. The CO_2_ and N_2_ gases were analysed using a Delta XP isotope-ratio mass spectrometer (Bremen, Germany) using a continuous flow system with every 20 unknowns separated by laboratory standards. In 2011 and 2012, samples were analysed at the Monash University Water Studies Centre, Australia, on an ANCA-GSL 2 elemental analyser. The resultant CO_2_ and N_2_ gases were analysed using a coupled Hydra 20:22 isotope-ratio mass spectrometer (Sercon Ltd., UK) with every five unknowns separated by laboratory standards. Sample precision was 0.1‰ for both *δ*^13^C and *δ*^15^N. Stable isotope abundances are expressed in *δ* notation in per mille units (‰) following the equation:

$$\delta^{13}\text{C or }\delta^{15}\text{N}=\left[\left(\frac{R_{\mathrm{sample}}}{R_{\mathrm{standard}}}\right)-1\right]\times 1000,$$

where *R*=(^13^C/^12^C or ^15^N/^14^N) of the sample and standards or where *R* is the ratio of the heavy (rare) isotope to the light (common) isotope in the sample and standard [60]. The international standards for carbon and nitrogen stable isotope ratios were Pee Dee Belemnite and atmospheric N_2_, respectively.

Inter-laboratory variability in stable isotope analysis of animal tissue can lead to discrepancies in *δ*^13^C/*δ*^15^N results between laboratories and care should be taken to ensure obtaining comparable outcomes [61]. In this study, replicate samples were not sent to both laboratories to ensure result congruency due to logistic, financial and ethical constraints. However, inter-laboratory stable isotope variability does not greatly influence this dietary reconstruction study for two reasons. Firstly, inter-laboratory stable isotope variability does not influence isotopic niche width. Therefore, within and between year changes in isotopic niche width are reflective of consumed prey and are not artefacts of inter-laboratory variability. Secondly, because penguin tissue and corresponding prey samples were processed at the same laboratory, dietary reconstruction models are controlled. However, the isotopic position of penguins and their prey could be influenced by inter-laboratory variability and caution should thus be exercised when comparing penguin isotopic positions between years 2007/2008 and 2011/2012.

### 3.6 Statistical analysis

All statistical analyses were performed using R software v. 2.14 [62]. Differences in stomach prey composition (of the three main prey species) between year (2007 and 2008) and life-stage (pre-moult: Feb/Mar/Apr; winter: May/June/Jul; breeding 2007: Sep/Oct/Nov, 2008: Aug/Sep/Oct) were tested using a two factor ANOVA with type III sums of squares. Life-stage and inter-annual differences in anchovy sizes were also determined using type III ANOVA. Feather and blood isotopic variations were corrected using regression equations [63], and corrected feather values were used in all statistical analyses. Differences in *δ*^13^C and *δ*^15^N between years and life-stages were tested using a two factor ANOVA with type III sums of squares to accommodate for the unbalanced sample design. For both *δ*^13^C and *δ*^15^N, a simple main effects test, using life-stage as a factorial subset, was analysed using MS_Resid_ from the global model. Tukey's post hoc tests were used to identify differences between life-stages for *δ*^13^C and *δ*^15^N. Differences in *δ*^13^C and *δ*^15^N between prey species (and between age classes) and across years were assessed using a multivariate analysis of variance (MANOVA).

Stable Isotope Analysis in R (SIAR) (v. 4.1.3) [64], a Bayesian computing framework, was used to solve mixing models. A non-informative Dirichlet prior distribution, with zero concentration dependencies, and default SIAR MCMC estimation (iterations=2×10^5^, burning=5×10^4^, thinning=15) were included in the model. Stable isotope mixing models were run for each year. Prey obtained in 2007/2008 was applied to mixing models in 2007 and 2008, and prey obtained in 2011/2012 was incorporated to 2011 and 2012 mixing models, respectively. An isotopic mean discrimination factor of 3.9‰ for *δ*^15^N and 0.2‰ for *δ*^13^C was applied to models, based on fractionation values obtained from little penguins experimentally fed a diet consisting solely of sprats (*Sprattus sprattus*) [65]. Prey proportion densities (50, 75 and 95% credibility intervals) for the pre-moult, winter and breeding life-stage in 2007, 2008, 2011 and 2012 were assessed to reconstruct the diet of penguins.

The SIAR function SIBER (stable isotope Bayesian ellipses in R) [22] was used to calculate the isotopic niche widths of pre-moult, winter and breeding birds from years 2007, 2008, 2011 and 2012. Standard ellipses represent the isotopic niche width of 40% (SIBER default) of typical individuals within the groups based on bivariate normal distributions. We used the corrected version of the standard ellipse area (SEAc) to account for the loss of an extra degree of freedom when calculating bivariate data and to control for small sample sizes [22]. A Bayesian estimate of the standard ellipse area (SEA_*B*_) was used to compare niche widths between groups. Differences in niche width between groups were compared in a probabilistic manner based on the size of simulated ellipse areas and their estimated posterior distributions [22]. Density plots display 50, 75 and 95% credibility intervals. Additionally, SIBER was used to calculate the isotopic niche widths of anchovy at three age cohorts (less than 1 year, 1–2 years and 2–3 years) to assess if this species displayed ontogenetic shifts in their niche width.

## 4. Results

### 4.1 Stomach content analysis

**Figure 1.**
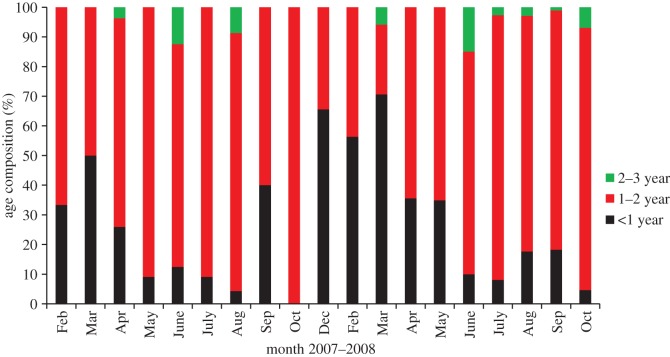
Age composition of anchovies recovered from little penguin stomach contents between February 2007 and October 2008. Age composition calculated from otolith size regression equations for standard length.

Based on the quantified mass of each prey item for each month, anchovy (*E. australis*) and southern garfish (*H. melanochir*) dominated the diet of penguins year-round (table 3). Cephalopods comprised only a small proportion of the diet overall with bay squid (*L. noctiluca*) being the most common species (table 3). The remaining species predominantly comprised Australian sprat (*Sprattus novaehollandiae*), blue sprat (*S. robustus*), sandy sprat (*H. vittatus*), pilchard (*S. sagax*) and hardyhead spp. The stomach content sampling showed no significant difference between year (*F*_1_=3.3, *p*>0.05) or life-stage (*F*_2_=2.3, *p*>0.05) for the three main species (anchovy, southern garfish and bay squid) consumed. Mean size of anchovy calculated from published otolith-standard length regression equations was approximately 72 mm, corresponding to anchovies of approximately 1–2-year age class. This age class dominated the diet of penguins year-round (figure 1). There was no significant difference in the size of anchovies taken either by year (*F*_1_=3.3, *p*>0.05, figure 1) or life-stage (*F*_2_=3.1, *p*>0.05, figure 1).

**Table 3.** Dietary contribution (weighted relative occurrence) of three main prey species identified in little penguin stomach contents between years 2007 and 2008. Samples were obtained from random individuals in time frames that broadly correspond with the pre-moult, winter and breeding life-stages of little penguins. Sample sizes only represent penguins from which stomach contents were obtained.

				% prey contribution by mass			
year	date stomachs sampled	corresponding life-stage	*n*	anchovy (*Engraulis*) (*australis*)	southern garfish (*Hyphorhamphus*) (*melanochir*)	bay squid (*Loliolus*) (*noctiluca*)	other
2007	Feb, Mar, Apr	pre-moult	15	55.5	3.9	17.5	23.1
2007	May, Jun, Jul	winter	19	83.6	8.0	6.2	2.2
2007	Sep, Oct, Nov	breeding	14	57.1	29.5	9.3	4.1
2008	Feb, Mar, Apr	pre-moult	21	77.8	8.6	8.5	5.1
2008	May, Jun, Jul	winter	20	72.5	17.4	6.8	3.3
2008	Aug, Sep, Oct	breeding	23	84.0	3.2	7.2	5.6

### 4.2 Penguin stable isotope ratios

A total of 440 blood and feather samples were obtained from penguins over the four years at three life-stages (pre-moult, winter and breeding) (table 1). The analysis of corrected isotope values revealed a significant interaction between year and life-stage for both *δ*^13^C signatures (*F*_6,429_=39.7, *p*<0.001, table 4) and *δ*^15^N signatures (*F*_6,429_=27.2, *p*<0.001, table 4). We observed inter-annual fluctuations in the isotopic niche position of penguins but found some consistent shifts in the isotopic position of penguins between life-stages. Within years, the winter isotopic position of penguins was consistently more enriched in *δ*^15^N and *δ*^13^C levels (excepting *δ*^13^C levels in 2007) compared with pre-moult birds (table 4). During the breeding season, the *δ*^13^C values of breeding penguins were generally more depleted than winter adults (except in 2007 when they did not differ significantly), while *δ*^15^N signatures shifted in an unpredictable manner between years (table 4).

**Table 4.** Differences in stable carbon (*δ*^13^C) and stable nitrogen (*δ*^15^N) isotope ratios between little penguin life-stages (pre-moult, winter and breeding) and years (2007, 2008, 2011 and 2012). Italicized values indicate significant differences in stable carbon and stable nitrogen isotope ratios between life-stages and years.

source	type III sum of squares	d.f.	mean square	*F*	*p*
*δ*^13^C					
life-stage	65	2		150.6	<*0.001*
year	11	3		17.4	<*0.001*
life-stage : year	52	6		39.7	<*0.001*
2007—life-stage	0.3	2	0.2	0.7	0.481
2008—life-stage	3.3	2	1.6	7.5	<*0.001*
2011—life-stage	88.6	2	44.3	204.2	<*0.001*
2012—life-stage	27	2	13.5	62.2	<*0.001*
resid	93	429	0.2		
*δ*^15^N					
life-stage	72	2		44.5	<*0.001*
year	243	3		99.9	<*0.001*
life-stage : year	133	6		27.2	<*0.001*
2007—life-stage	28.1	2	14.1	17.3	<*0.001*
2008—life-stage	6.8	2	3.4	4.2	*0.0156*
2011—life-stage	84	2	42	51.7	<*0.001*
2012—life-stage	85.3	2	42.7	52.3	<*0.001*
resid	348	429	0.8		

### 4.3 Prey stable isotope ratios

In 2007/2008, the *δ*^13^C and *δ*^15^N isotopic values of prey differed significantly between species (*δ*^13^C: *F*_2,41_=39.8, *p*<0.001, *δ*^15^N: *F*_2,41_=11.3, *p*<0.001). The stable isotope values of prey ranged from −21.5 to −15.2‰ for normalized *δ*^13^C and from 20.2 to 13.8‰ for *δ*^15^N. Anchovy had the most depleted mean *δ*^13^C levels, while southern garfish was most enriched (table 2). Bay squid was the most enriched in *δ*^15^N levels between species (table 2). The isotopic values of anchovies differed significantly between ontogenetic stages for normalized *δ*^13^C values (*F*_2,20_=4.9, *p*<0.05) but not for *δ*^15^N (*F*_2,20_=1.62, *p*>0.05). Anchovies in the 2–3 year cohort had the most depleted mean *δ*^13^C values (−20.7±0.5‰) and those in the 1–2 year cohort had the most enriched mean *δ*^13^C levels (−20.3±0.1‰). Due to the lack of *δ*^15^N isotopic position distinction between ontogenetic stages, all anchovy results were pooled for stable isotope mixing models.

In 2011/2012, the stable isotope values of prey ranged from −21.7 to −14.6‰ for *δ*^13^C and from 20.2 to 10.9‰ for *δ*^15^N. Significant differences among species (and between juvenile and adult pilchard) for *δ*^13^C (*F*_5,51_=205.6, *p*<0.001) and *δ*^15^N (*F*_5,51_=136.8, *p*<0.001) were found. Southern garfish had the most enriched mean *δ*^13^C value, whereas blue sprat had the most depleted mean *δ*^13^C value (table 2). Anchovy displayed the highest mean *δ*^15^N signature and juvenile pilchard had the most depleted mean *δ*^15^N signature (table 2).

We found significant differences in the stable isotope composition of anchovy between years 2007/2008 and 2011/2012 for *δ*^13^C (*F*_1,34_=262.7, *p*<0.001) and *δ*^15^N (*F*_1,34_=50.9, *p*<0.001). In 2011/2012, anchovies were more enriched in both *δ*^13^C (2.4‰) and *δ*^15^N (2.8‰) compared with anchovies 2007/2008. In 2011/2012, southern garfish was more enriched in *δ*^13^C compared with garfish in 2007/2008 (*F*_2,21_=3.7, *p*<0.005). No significant difference in *δ*^15^N levels was found. Our results show that annual variations in the isotopic niche of penguins are influenced by inter-annual changes in the stable isotopic composition of prey.

### 4.4 Stable isotope mixing models

**Figure 2.**
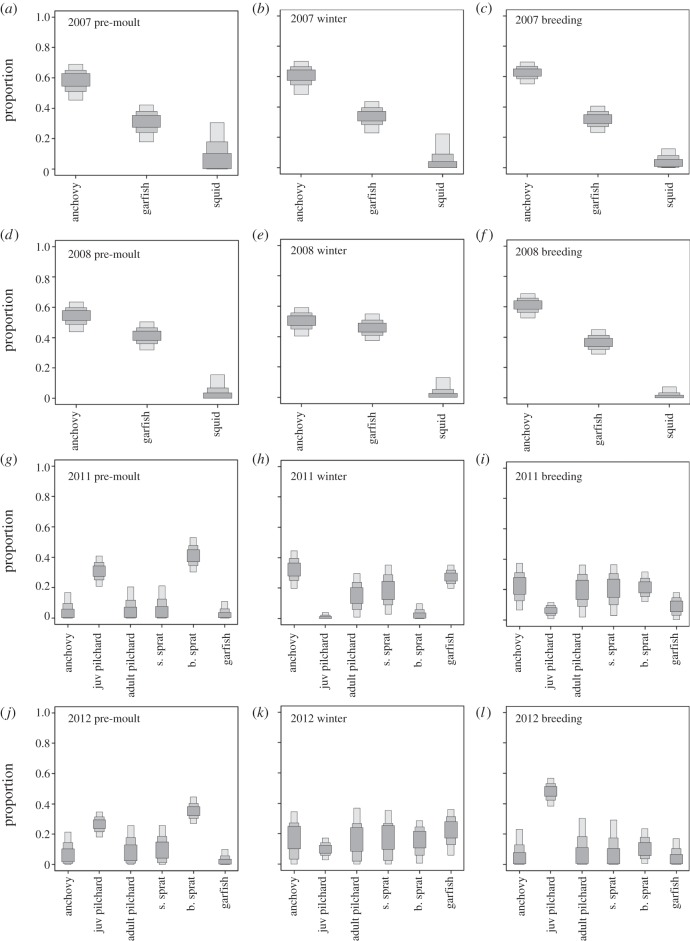
(*a*–*l*) Stable isotope mixing model estimated prey source contributions to the pre-moult, winter and breeding diet of little penguins in 2007, 2008, 2011 and 2012 (±95, 75 and 50% credibility intervals). s. sprat, sandy sprat; b. sprat, blue sprat.

Stable isotope mixing model outputs revealed significant differences in the relative proportion of ingested food sources between years. In 2007, the diet of penguins was dominated by anchovies in all life-stages (figure 2*a*–*c*). Southern garfish contributed significantly to penguin diet, and bay squid contributed least among resources. Compared to 2007, the relative contribution of anchovy to penguin diet declined in 2008, but anchovy continued to dominate the diet of penguins in all life-stages (figure 2*d*–*f*). An increase in the contribution of southern garfish to penguin diet was observed and bay squid continued to contribute little over the entire course of the year.

In 2011 anchovy's contribution to penguin diet decreased in all life-stages compared with years 2007 and 2008 (figure 2*g*–*i*). Blue sprat and pilchard dominated the pre-moult diet of penguins and sandy sprat, anchovy and southern garfish contributed marginally. During winter, anchovy's contribution to penguin diet increased and it had a mean proportional contribution of 32%, followed by southern garfish and sandy sprat. The contribution of pilchard and blue sprat to penguin diet was minor. During the 2011 breeding season, penguins displayed a diverse diet with similar contributions of anchovy, sandy sprat, blue sprat and pilchard. Garfish was detected at relatively low levels.

In 2012, anchovy's contribution to penguin diet was the lowest among all years of the study (figure 2*j*–*l*). During pre-moult, the diet of penguins was dominated by blue sprat (35%) and pilchard (37%). The remaining prey sources displayed a mean proportional contribution between 3 and 12%. During winter, the diet of penguins was dominated by southern garfish, which had a mean proportional contribution of 21%. The remaining prey sources contributed similar quantities to penguin diet with mean proportional contributions between 10 and 18%. During the 2012 breeding season, the diet of penguins was dominated by pilchard, which had a mean proportional contribution of 59%. The remaining species contributed similarly with mean proportional contributions between 7 and 12%.

### 4.5 Inter- and intra-annual isotopic niche variation

**Figure 3.**
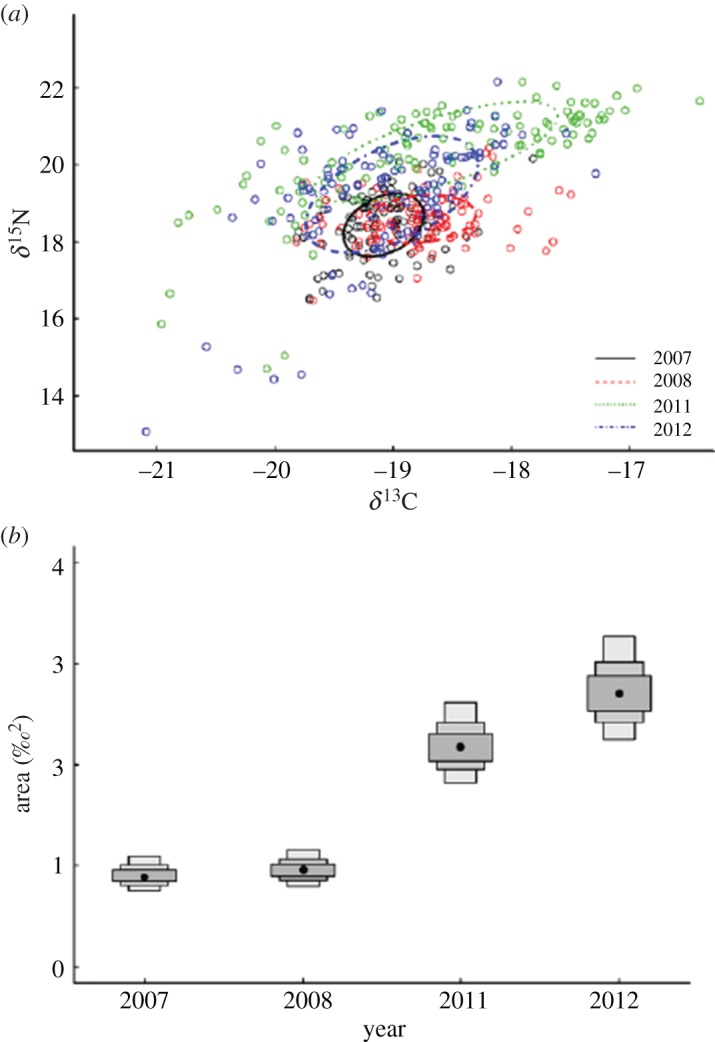
(*a*) Biplots depicting the overall annual *δ*^13^C and *δ*^15^N isotope ratios of little penguins in years 2007, 2008, 2011 and 2012. Ellipses represent the isotopic niche width of 40% of typical individuals within the group based on bivariate normal distributions. (*b*) Density plots depict the mean ellipse areas (represented by black dots) and their credibility intervals (50, 75 and 95%). The degree of overlap in credibility intervals between years is indicative of the degree of similarity in isotopic niche width between groups.

Over the four year period, we observed a large degree of isotopic niche overlap (SEAc, figure 3*a*), particularly between years 2007/2008 and 2011/2012. SIBER analysis revealed that the overall isotopic niche width (SEA_*B*_) of penguins in 2007 and 2008 did not differ significantly in size (figure 3*b*). Their isotopic niche widths were significantly narrower than penguins in 2011 and 2012. The isotopic niche width of penguins in 2011 and 2012 did not differ significantly.

**Figure 4.**
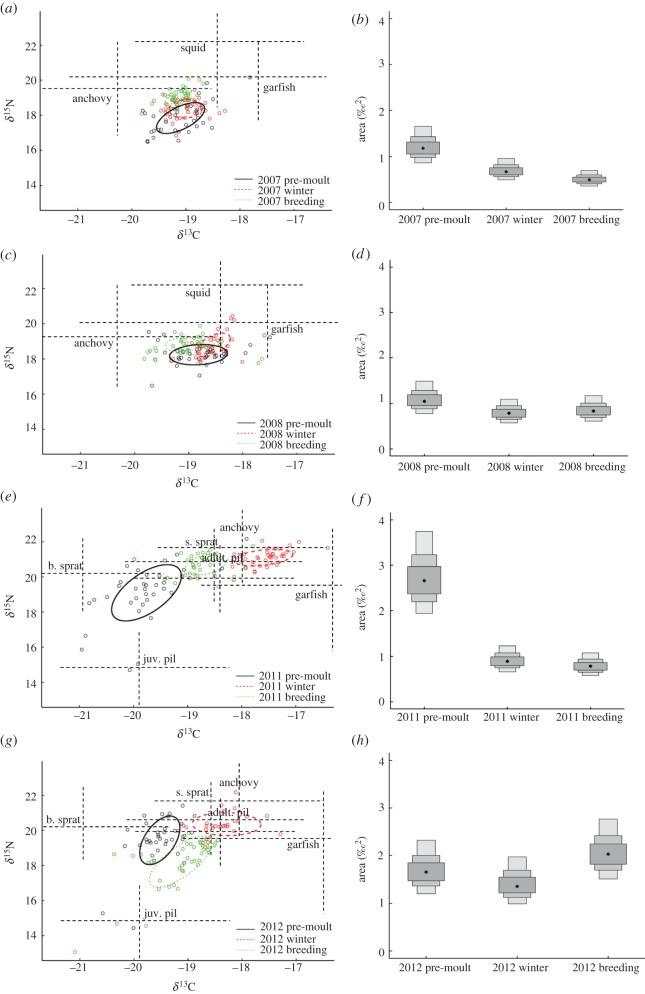
(*a*–*h*) Dotted lines within biplots represent the isotopic position of penguin prey (±s.e.). Ellipses represent the *δ*^13^C and *δ*^15^N isotope ratios of pre-moult, winter and breeding life-stages over four years and their corresponding density plots which depict the mean standard ellipse areas (represented by black dots) and their credibility intervals (50, 75 and 95%). s. sprat, sandy sprat; b. sprat, blue sprat.

In 2007, a strong overlap in the isotopic niche of pre-moult and winter adults was found (figure 4*a*). The isotopic niche width of pre-moult penguins was wider than both winter and breeding adults, while the niche widths of winter and breeding adults were comparable in size (figure 4*b*). In 2008, there was a high degree of isotopic niche overlap between all life-stages (figure 4*c*) and the isotopic niche width of pre-moult, winter and breeding penguins did not differ substantially (figure 4*d*). In 2011, no isotopic niche area overlap was observed between life-stages (figure 4*e*). The isotopic niche width of pre-moult penguins was the widest between life-stages, while winter and breeding birds displayed no difference in niche width (figure 4*f*). In 2012, apart from a minor overlap in the isotopic niche area between pre-moult and breeding penguins (1% and 0.8%, respectively) no isotopic niche area overlap was observed (figure 4*g*). No difference in isotopic niche width between life-stages was evident (figure 4*h*).

**Figure 5.**
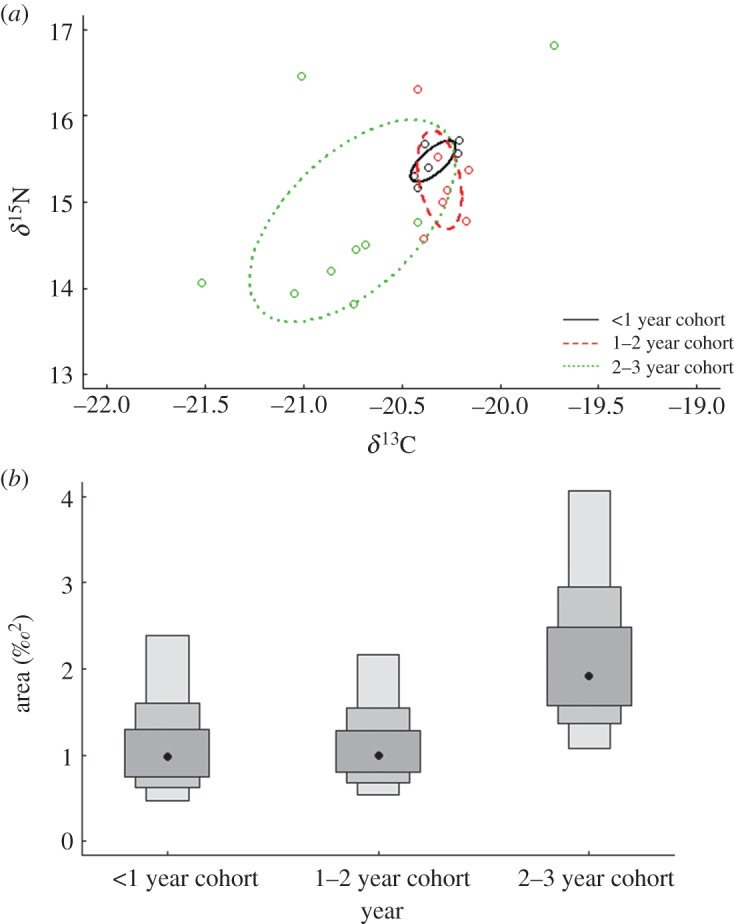
(*a*) Biplot depicting the *δ*^13^C and *δ*^15^N isotope ratios of anchovies at three ontogenetic stages. (*b*) Density plots depict the mean standard ellipse areas (represented by black dots) and their credibility intervals (50, 75 and 95%). The degree of overlap in credibility intervals between cohorts is indicative of the degree of similarity in isotopic niche width between groups.

There was some degree of isotopic niche overlap between all anchovy ontogenetic stages (figure 5*a*) and no difference in the isotopic niche width of the groups was found (figure 5*b*).

## 5. Discussion

### 5.1 Inter-annual diet and isotopic niche

Anchovy dominated the diet of penguins across years, confirming previous dietary studies at this colony [30,31]. The inter-annual dominance of anchovy in penguin diet indicates these predators inhabit an environment that contains a relatively predictable prey resource. Indeed, the bay provides an important spawning ground for mature anchovy and their peak abundance coincides with the breeding period of little penguins [66,67]. Additionally, larvae and juvenile anchovy use the bay as a nursery and are available in Port Phillip Bay throughout the year [67,68]. However, the relative abundance of anchovy in Port Phillip Bay fluctuates significantly between years [53], and these fluctuations are reflected in the variable contribution of anchovy to the stomach content, reconstructed diet as well as the isotopic niche shifts of penguins.

We observed fluctuations in the isotopic niche position and isotopic niche width of penguins between years. Inter-annual fluctuations in consumed prey are particularly evident between years 2007/2008 and 2011/2012. Based on independent fish surveys that were conducted in Port Phillip Bay between years 2008 and 2011, pilchard, sandy sprat and blue sprat were significantly more abundant in 2011 compared with years 2008–2010 [53]. The increased contribution of these prey taxa to penguin diet in 2011/2012 are reflected in their broad isotopic niche widths and highlight the flexible foraging strategies of penguins in response to fluctuations in prey resources.

### 5.2 Life-stage variation in diet and isotopic niche

#### 5.2.1 Pre-moult

Prior to moult, little penguins undertake an intensive foraging bout that lasts approximately three weeks. During this time, they can almost double their mean body mass to provide the energy required for a fast that lasts approximately 17 days [34]. For all penguin species, moulting is an energetically demanding process [1,69] and accounts for approximately 8.4% of the annual energy budget in little penguins [35]. Access to abundant prey during the three-week foraging bout is vital to ensuring penguins have the energetic resources to undergo a complete moult, which is critical to their survival and breeding success [70,71]. We found the isotopic niche position and isotopic niche width of pre-moult penguins varied widely between years, particularly between years 2007/2008 and 2011/2012. Similarly, the pre-moult stomach content (2007 and 2008) and reconstructed diet of penguins varied between years. This implies resources obtained at this life-stage may be less predictable compared with other stages of the annual cycle and that pre-moult penguins may be exposed to particularly variable prey conditions. Declines in local prey abundance towards the end of the breeding season (a period coinciding with the pre-moult stage of a large proportion of little penguins in this colony) have been recorded within the home ranges of several central place foraging species [72,73], including the little penguin [28,74]. Because pre-moult penguins are not constrained by breeding activities, including the need to alternate incubation shifts or feed chicks regularly, they can overcome local fluctuations in prey by increasing their foraging effort (e.g. extending foraging range, duration) with no expense to the survival of their young. Therefore, the timing of moult may occur when resources are less predictable compared to other stages of the year. As adults are not constrained by breeding demands, they can more readily overcome resource limitations they otherwise face during the breeding season.

The observed differences in the isotopic niche position and niche width of penguins in 2007/2008 and 2011/2012 may also be related to differences in the turnover rates of penguin tissues (i.e. blood (2007/2008) versus feathers (2011/2012)). Feathers tend to have higher *δ*^13^C and *δ*^15^N isotopic values than blood, even when synthesized over the same temporal scale, due to tissue-specific discrimination factors [63]. We used corrected feather values to account for this discrepancy. But this mathematical correction does not account for the potential influence of fasting which can induce greater levels of *δ*^13^C and *δ*^15^N enrichment on certain tissues over others [75]. Further research aimed at identifying metabolic pathways in moulting little penguins is necessary to confirm pre-moult dietary shifts in this penguin colony.

#### 5.2.2 Winter

During winter, anchovies dominated the stomach content (2007 and 2008) and reconstructed diet of penguins in all years except 2012. This finding is supported by studies that document that anchovies constitute the majority of the clupeoid population biomass in winter [66,67]. Additionally, penguins were consistently more enriched in *δ*^15^N and *δ*^13^C levels (excepting *δ*^13^C levels in 2007) relative to pre-moult birds. Stable isotope mixing models signal enriched *δ*^15^N and *δ*^13^C levels were in part due to the increased consumption of southern garfish (2007, 2008) and anchovy (2011, 2012). However, we propose that enriched *δ*^15^N and *δ*^13^C levels were in response to the increased consumption of juvenile anchovy (less than 1 year and 1–2 year cohort) that are reported to constitute the majority of the Port Phillip Bay anchovy biomass in winter [66,67]. Juvenile anchovies are significantly more enriched in *δ*^13^C and appear to be more enriched in *δ*^15^N levels relative to anchovy adults (2–3 year cohort) (figure 5*a*), and the increased consumption of juvenile cohorts may account for the consistent enrichment in *δ*^15^N and *δ*^13^C levels in winter penguins. Mixing models could not confirm the increased contribution of juvenile anchovies to penguin diet because all three anchovy age cohorts were aggregated in stable isotope mixing models (due to indistinct *δ*^15^N values between age cohorts) [76]. Similarly, stomach content results in the winters of 2007 and 2008 demonstrated that even though anchovy dominated the diet of penguins, there were no obvious seasonal increases of less than 1 year and 1–2 year anchovy cohorts in winter compared with other times of the year. The methodological constraints of each dietary sampling technique differ [77], making it difficult to determine whether ontogenetic dietary shifts are driving the consistent isotopic trends in the winter diet of penguins. One way to overcome these limitations would be to include distinct ontogenetic stages of prey as discrete nodes in food-web models. Ontogenetic shifts in the niche position or niche width of prey will have flow on effects on the isotopic dimensions of predators [78], and through including several ontogenetic stages in food-web models, dietary studies could be better able to identify prey resources critical to the survival of seabirds at various life-stages. The nutritional importance of different ontogenetic stages of particular prey species have been documented for several seabird species (e.g. black-legged kittiwakes, *Rissa tridactyla*) [79], and knowledge of these prey types is vital to the conservation of seabirds and their prey. The ongoing improvement of stable isotope mixing models may eventually increase the research capacity to discriminate between sources that are not entirely distinct.

#### 5.2.3 Breeding

During the breeding season, the *δ*^13^C values of penguins were more depleted than winter adults (except in 2007 when they did not differ significantly). Usually, depleted *δ*^13^C values are an indication that seabirds are foraging in offshore areas [19,48,80]. However, during the breeding season, little penguins remain inshore within 20 km of their breeding colony [27,29]. Thus, in this study, depleted *δ*^13^C values probably reflect the increased consumption of prey entering Port Phillip Bay from offshore regions. During the austral spring and summer, a variety of prey species, including adult anchovy, enter Port Phillip Bay from offshore waters to spawn [32,55,81]. Additionally, high numbers of juvenile fish (including 0+, 1+ year class pilchards) enter the bay from offshore waters and use the area as a nursery [55]. This influx of fish from offshore regions is probably responsible for the depleted *δ*^13^C levels in breeding penguins. Stomach content analyses over summer months in 2007 and 2008 found no seasonal increase in the adult anchovy cohort. But, the stomach content study found an increased abundance of Australian sprat, sandy and blue sprat which are thought to enter bays and inlets in southwestern Australia to spawn in spring and summer months [81–83], providing support for the tenet that depleted *δ*^13^C values reflect the increased consumption of prey entering Port Phillip Bay from offshore regions.

Within Port Phillip Bay, the peak spawning activity of anchovy occurs in mid-summer [32,66], coinciding with the breeding season of little penguins. We found anchovies dominated the stomach content and reconstructed diet of breeding penguins in 2007, 2008 and 2011, but because the relative abundance of this (and other) species fluctuates significantly between years [53], the breeding diet, isotopic niche width and isotopic niche position of little penguins varied between years. The variations in penguin diet and isotopic niche demonstrate their ability to modify their diet to accommodate fluctuations in prey. This foraging strategy is critical to St Kilda penguins, which have a small foraging range (in terms of depth and distance) [84] and need to maximize resource intake to reproduce successfully.

At St Kilda, the onset of the breeding season is highly variable from year to year [31,85] and little penguins may time breeding activities to coincide with increased resource abundance in Port Phillip Bay. This reproductive strategy is in line with the reproductive ecology of several seabird species who time reproduction with peak prey abundance, thus matching peak food demands with peak prey abundance, thereby increasing their reproductive success [86,87]. Despite the ability of little penguins to adjust their breeding date to coincide with increased resource abundance [42] and modify their diet to maximize resource intake, they display high variability in their annual reproductive success [39,85]. Consequently, the continued monitoring of their diet and reproductive parameters will be central to their ongoing management. Monitoring the breeding diet, isotopic niche widths and breeding parameters of seabirds can be used to gauge the diversity and abundance of prey available during the breeding season [85]. For example, poor reproductive success in association with broad diets and niche widths can indicate poor foraging conditions, where declines in preferred prey species force penguins to resort to less ‘favoured’ prey, and expand their dietary niche [88,89]. Alternatively, poor reproductive success in association with narrow dietary diversity and narrow niche widths can be indicative of constrained foraging conditions. The continued decline of annual reproductive success in association with decreasing dietary breadth and niche widths can be indicative of food-web structure simplification [90,91]. Moreover, unlike migratory seabirds that disperse widely after the breeding season, resident seabird species' diets, demographics and body condition indices can be monitored year-round to provide within and between year assessments of local prey composition and availability [92]. This information can be pertinent in determining if niche width collapse is imminent in marine systems like bays and estuaries that are often subjected to large-scale human impact [93,94].

## 6. Conclusion

The primary goal of this study was to assess how the diet of a resident inshore seabird varies across the annual cycle and to identify the key prey resources of little penguins. We propose that during the non-breeding season, penguins target juvenile fish communities, particularly juvenile anchovy, which use the bay as a nursery and dominate the prey biomass in winter months [67,68]. During the breeding season, penguins appear to exploit prey species that enter Port Phillip Bay from offshore waters to spawn [68,95]. Due to this year-round availability of prey in the bay, little penguins can remain within close proximity to their breeding area at all stages of the annual cycle, which may have energetic benefits that ultimately improve their long-term reproductive success [92]. Additionally, our study demonstrates that although the diet of little penguins is dominated by anchovy year-round, they can switch between prey types in response to fluctuations in prey availability. Therefore, rather than ensuring specific prey stocks are available to penguins, it is important to identify and protect the environmental features that attract and sustain spawning and juvenile fish communities in this highly urbanized semi-enclosed embayment. For example, seagrass habitats within the bay are highly productive systems offering food and shelter for a rich assemblage of fish communities in addition to stabilizing other ecosystem functions [96]. But, their cover has declined in many areas within the bay and further research is required to determine the factors driving their decline to ensure the ongoing recruitment success of many clupeoid species [96]. In the meantime, monitoring penguin diet, relative position in *α*-space (stable isotope analysis), and indices of their year-round body condition and reproductive success will be a useful indicator of the viability of penguins as well as local foraging conditions.

## Supplementary Material

Stable isotope and stomach content analyses values for little penguins (Eudyptula minor)

## Data accessibility

See the electronic supplementary material to obtain data used in this paper.
